# Effects and Mechanisms of Attapulgite Clay-g-(AA-co-AAm) Hydrogel (ACH) in Alleviating Saline Stress in Spinach

**DOI:** 10.3390/plants14213330

**Published:** 2025-10-31

**Authors:** Yinhua Wang, Bingqin Teng, Haodong Zhang, Zhengqian Zhou, Yangbin Xin, Liqun Cai, Jun Wu

**Affiliations:** 1 College of Resources and Environment, Gansu Agricultural University, Lanzhou 730070, China; 19944159607@outlook.com (Y.W.); bingqinteng@outlook.com (B.T.); cnzhhd@163.com (H.Z.); 18294897803@163.com (Z.Z.); xinyangbin11@163.com (Y.X.); cailq@gsau.edu.cn (L.C.); 2 State Key Laboratory of Aridland Crop Science, Gansu Agricultural University, Lanzhou 730070, China

**Keywords:** growth indicators, physiological regulation, ion balance, osmotic regulatory substances

## Abstract

Soil salinization restricts the sustainable development of global agriculture, expanding at an annual rate of approximately 1 million hectares. In China, the total area of saline–alkali land reaches 170 million hectares, of which the arable land area exceeds 50 million hectares. The arid northwest region witnesses worsening soil salinization due to arid climate and improper irrigation practices, which seriously affects the yield of crops such as spinach (*Spinacia oleracea* L.). As a leafy vegetable with high nutritional value and economic significance, spinach exhibits growth inhibition, leaf yellowing, and disrupted physiological metabolism under saline–alkali stress. Therefore, this study investigates the alleviating effects and mechanisms of Attapulgite Clay-g-(AA-co-AAm) Hydrogel (ACH) on spinach under salt stress (NaCl) and alkaline stress (NaHCO_3_). The results show that ACH has a loose, porous structure. As the addition of Attapulgite Clay increases, the surface roughness and porosity improve while retaining organic functional groups (amide groups, carboxyl groups) and inorganic Si-O bonds, providing a structural foundation for stress mitigation. In terms of yield enhancement, ACH effectively alleviates salt–alkali stress: under severe salt stress (SS2), 0.2% ACH increased leaf area by 91% and leaf weight by 95.69%; under mild alkaline stress (AS1), 0.2% ACH increased leaf area by 46.3% and leaf weight by 46.21%; and under severe mixed salt–alkali stress (MS2), 0.4% ACH increased root weight by 49.83%. Physiologically, ACH reduced proline content (51.25% reduction under severe mixed stress) and malondialdehyde (MDA) content (68.98% reduction under severe alkaline stress) while increasing soluble sugar content (63.54% increase under mixed stress) and antioxidant enzyme activity (SOD, POD, CAT). In terms of ion regulation, ACH reduced Na^+^ accumulation in roots and leaves (61.12% reduction in roots and 36.4% reduction in leaves under severe salt stress) and maintained potassium–sodium balance. To conclude, ACH mitigates the adverse effects of salt–alkali stress by coordinately modulating spinach’s growth, physiological metabolic processes, and ion balance. This synergistic regulatory effect ultimately contributes to sustaining high yields of spinach.

## 1. Introduction

Soil salinization is a major environmental issue constraining the sustainable development of global agriculture [1,2], expanding at a rate of approximately 1 million hectares per year. China has a total area of 170 million hectares of saline–alkali land, of which over 50 million hectares are arable saline–alkali land suitable for agricultural [3]. The northwestern region of China, as an important dryland agricultural area, faces increasingly severe soil salinization issues due to factors such as arid climate and improper irrigation, leading to reduced crop yields and quality and posing a serious threat to regional food security and ecological stability [4]. Spinach (*Spinacia oleracea* L.), a widely cultivated leafy vegetable rich in nutrients with high economic and edible value, is significantly inhibited in its growth and development by saline–alkali stress, manifesting as leaf yellowing, reduced biomass, impaired photosynthesis, and disrupted physiological metabolism [5]. Therefore, exploring effective technical measures to alleviate spinach salt–alkali stress is of great significance for improving spinach yield and quality in saline–alkali regions and promoting efficient agricultural utilization of saline–alkali land.

Attapulgite Clay, a natural nano-sized clay mineral with a unique one-dimensional rod-like crystal structure and abundant surface active groups, is a common raw material for composite hydrogels. Attapulgite Clay-g-(AA-co-AAm) Hydrogel (ACH) combines the hydrophilicity of organic polymers and the stability of inorganic clays, showing significant potential in soil improvement and ion adsorption [6,7]. Investigating ACH’s effects and mechanisms in alleviating spinach salt–alkali stress can support saline–alkali land improvement and sustainable agriculture. Wu et al. [6] showed that attapulgite composite hydrogels have better adsorption via their porous structure; Li et al. [8] found that montmorillonite–acrylamide hydrogen bonding boosts hydrogel stability, aiding organic-inorganic synergy study. Regarding the impact of hydrogels on crop growth, Nassaj et al. [5] showed that clay-composite hydrogels increased tomato leaf area by 82% under salt stress via better rhizosphere water/nutrient absorption. Chen et al. [9] proposed that hydrogels’ effect in alleviating crop growth under alkaline stress is concentration-dependent, and low-concentration ones work more effectively by regulating the soil’s pH buffering capacity. Kant et al. [10] and Hu B et al. [11] noted that hydrogels reduce proline accumulation and activate SOD/POD. Additionally, Pathak et al. [12] proposed that hydrogels maintain cellular osmotic pressure by promoting soluble sugar synthesis. Zha et al. [13] found that montmorillonite hydrogels fix soil Na^+^; Fang et al. [14] showed that clay hydrogels promote K^+^ transport. However, most studies have focused on single stresses. Further investigation is needed into the synergistic regulatory mechanisms of mixed saline–alkali stress and ACH on spinach’s physiological metabolism and ion homeostasis.

To address existing research limitations, this study focuses on Attapulgite Clay-g-(AA-co-AAm) Hydrogel (ACH) and spinach under saline–alkali stress, investigating ACH’s mitigating effects under salt stress (NaCl), alkaline stress (NaHCO_3_), and mixed salt–alkaline stress (NaCl + NaHCO_3_). The study characterizes ACH’s microstructure/functional groups via SEM and IR spectroscopy and analyzes Attapulgite Clay addition’s impact on hydrogel porosity and organic–inorganic synergy. This research also measures spinach’s growth indicators (leaf area, biomass) and physiological parameters (chlorophyll, proline, soluble sugars, antioxidant enzymes) to reveal ACH’s role in improving photosynthesis and stress resistance, and analyzes Na^+^/K^+^ changes in roots/leaves to confirm its effect on maintaining K^+^-Na^+^ balance via ion homeostasis. The study aims to elucidate ACH’s comprehensive mitigation mechanism, providing a reference for high-yield, high-quality vegetable cultivation.

## 2. Results

### 2.1. Scanning Electron Microscopy (SEM)

**Figure 1:**
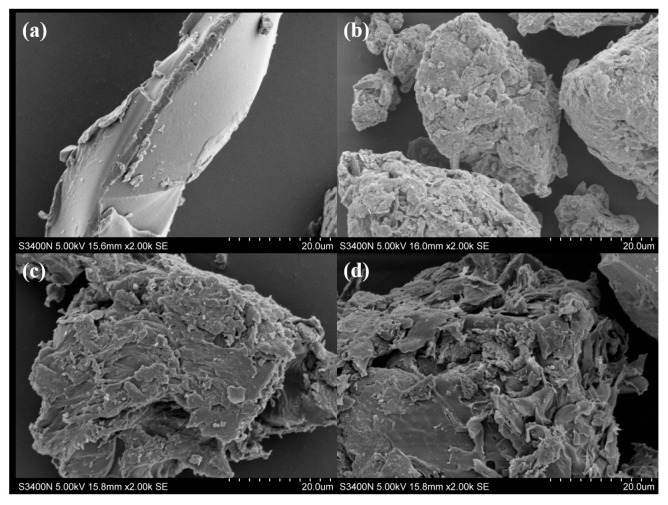
Scanning electron microscope analysis. CK (**a**), AC (**b**), ACH-1 (**c**), and ACH-2 (**d**).

As shown in Figure 1a, the AA-co-AAm hydrogel exhibits a relatively regular structure with high surface smoothness, indicating that after polymerization, the molecular chains of the AA-co-AAm hydrogel form a dense, low-porosity structure with a smooth surface through covalent cross-linking. Figure 1b shows the microstructure of Attapulgite Clay. Attapulgite Clay is a natural nanoclay with a one-dimensional nanorod crystal structure, forming loose aggregates rather than a chemically cross-linked polymer network. The nanorods interlock but remain independent particles with strong dispersion. Figure 1c,d show the Attapulgite Clay-g-(AA-co-AAm) Hydrogel (ACH), which exhibits significant morphological differences compared to the pure polymer hydrogel in (a) and the pure Attapulgite Clay in (b), presenting a more loose, porous, and rough block-like/aggregate-like structure. This is attributed to the participation of Attapulgite Clay in the graft copolymerization process: the active sites on the surface of its nanorod crystals (e.g., hydroxyl groups) undergo graft reactions with AA and AAm molecular chains, incorporating inorganic components into the polymer network, ultimately forming a multi-interface, porous three-dimensional network through the synergistic interaction between polymers and Attapulgite Clay. It is worth noting that in (c) and (d), the difference stems from the varying addition amounts of Attapulgite Clay (AC): the addition amount in (c) is relatively low, accounting for 2.5% of the total mass, while that in (d) is higher, making up 5% of the total mass. The microstructural morphology exhibits differences: compared to (c), (d) shows a higher surface roughness and more abundant and developed pores. This clearly demonstrates that when the Attapulgite Clay addition is 5%, its shaping effect on the “porous and rough” microstructure of the hydrogel is stronger.

### 2.2. ACH Surface Functional Groups

**Figure 2:**
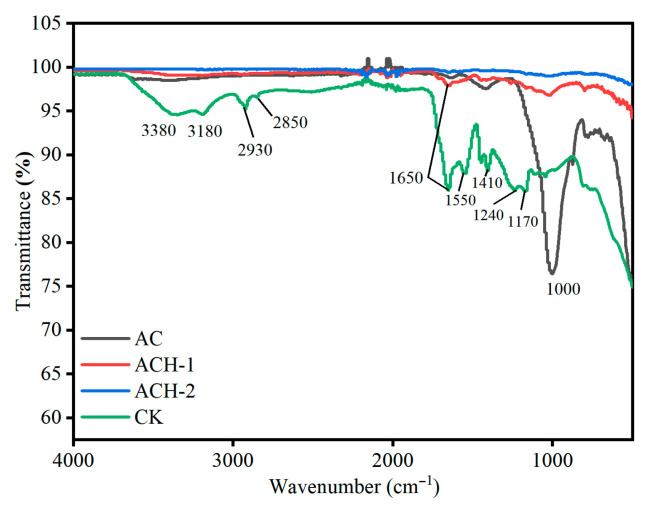
Fourier Transform Infrared (FTIR) spectra. The figure compares Attapulgite Clay (AC), pure AA-co-AAm hydrogel (CK), and Attapulgite Clay-g-(AA-co-AAm) Hydrogels with different Attapulgite Clay addition levels (ACH-1: 2.5% addition; ACH-2: 5% addition).

For AC (Attapulgite Clay), 1000–1200 cm^−1^ is attributed to Si-O and other inorganic bond vibration peaks, reflecting the mineral framework. For CK (AA-co-AAm Hydrogel), 3380 and 3180 cm^−1^ correspond to amide N-H and carboxyl O-H stretching vibrations; 2850 and 2930 cm^−1^ are alkyl C-H stretching vibrations; 1650, 1550, and 1410 cm^−1^ correspond to amide C=O stretching (amide I band), N-H bending (amide II band), and C-N stretching (amide III band); and 1770 cm^−1^ corresponds to the C=O stretching vibrations of the carboxyl group, while 1240 and 1000 cm^−1^ are associated with C-O stretching vibrations of the carboxyl group and ether bonds, reflecting their organic functional group characteristics. ACH-1 and ACH-2 (Attapulgite Clay-g-(AA-co-AAm) Hydrogel) retain the characteristic functional group vibration peaks of CK, but the 3380 and 3180 cm^−1^ (hydrogen-containing functional groups), 2850 and 2930 cm^−1^ (alkyl C-H), 1650–1410 cm^−1^ (amide groups), and 1770, 1240, and 1000 cm^−1^ (carboxyl-related) peaks differ in shape and intensity from CK, due to the grafting of Attapulgite Clay altering the functional group environment. Additionally, ACH-1 and ACH-2 exhibit peak shape differences reflecting adjustments in the functional group environment with varying inorganic phase content. This is manifested as subtle shifts in the positions of characteristic peaks, accompanied by corresponding changes in peak intensity. Spectroscopically, this directly reflects the regulation of the chemical environment of functional groups within the hydrogel by variations in inorganic phase content, with spectral parameters such as peak position, intensity, and width exhibiting regular changes, depending on the amount of Attapulgite Clay added (see Figure 2).

### 2.3. Effect of ACH Addition on Growth Indices of Spinach

#### 2.3.1. Leaf Parameters

**Figure 3:**
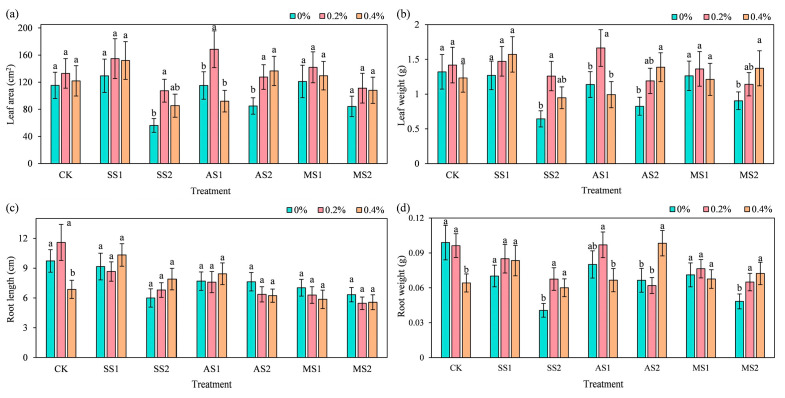
Effects of adding ACH combined with SS, AS, and MS on the morphological and growth indices of spinach. (Note: SS1 refers to mild saline–alkali stress; SS2 refers to severe salt stress; AS1 refers to mild alkali stress; AS2 refers to severe alkali stress; MS1 refers to mild mixed saline–alkali stress; MS2 refers to severe mixed saline–alkali stress). In the figure, lowercase letters indicate statistically significant differences between different ACH addition groups (*p* < 0.05). (**a**) Leaf area; (**b**) Leaf weight; (**c**) Root length; (**d**) Root weight.

As shown in Figure 3a, under SS2 treatment (severe salt stress), the addition of 0.2% ACH significantly increased leaf area by 91% (*p* < 0.05) compared to no ACH addition, indicating that ACH can effectively alleviate the inhibitory effect of severe salt stress on leaf area expansion. In the AS1 treatment group (mild alkaline stress), the addition of 0.2% ACH resulted in a significant increase of 46.3% in leaf area compared to the group without ACH (*p* < 0.05). This suggests that ACH can enhance the photosynthetic potential of plants under salt and alkaline stress by increasing leaf area, and its alleviating effect on severe salt stress is superior to that on mild alkaline stress. As shown in Figure 3b, in the AS1 treatment group, adding 0.2% ACH significantly increased leaf weight by 46.21% compared to adding 0% ACH. Under severe stress treatments (SS2, AS2, MS2), the addition of ACH significantly increased leaf weight, with the most pronounced effect observed in the SS2 treatment group, where 0.2% ACH increased leaf weight by 95.69% (*p* < 0.05). This indicates that ACH can enhance plants’ ability to accumulate substances under stress conditions by increasing leaf biomass (leaf weight), and its promotional effect on leaf weight is particularly pronounced under severe salt stress.

#### 2.3.2. Root Parameters

As shown in Figure 3c, in the CK treatment group, adding 0.4% ACH resulted in a significant decrease of 29.45% in root length compared to adding 0% ACH (*p* < 0.05). Overall, after stress application, adding ACH had no significant effect on spinach root length. In the AS2 treatment group (severe alkaline stress), the addition of 0.4% ACH resulted in a significant increase of 47.99% in root weight compared to the addition of 0% ACH. In the MS2 treatment group (severe mixed salt–alkaline stress), the addition of 0.4% ACH resulted in a significant increase of 49.83% in root weight compared to the addition of 0% ACH.

**Figure 4:**
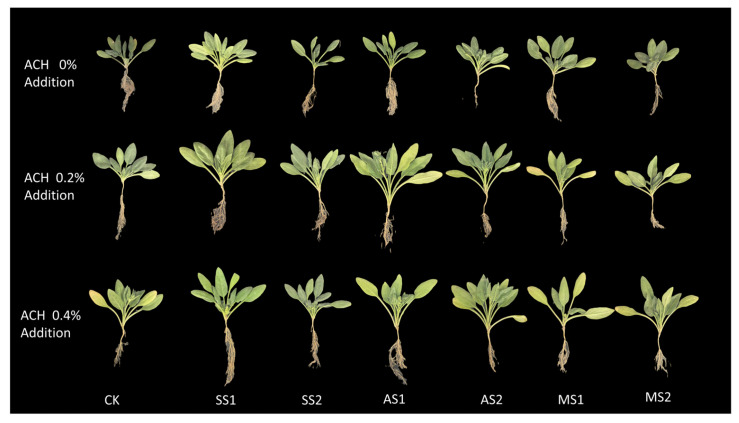
Attapulgite Clay-graft-(Acrylic Acid-co-Acrylamide) Hydrogel (ACH) exerts a mitigating effect on spinach under salt–alkali stress. The figure displays the most representative photographs of spinach plants from three repeated treatments.

As shown in Figure 4, under severe salt stress (SS2), the leaf area of spinach in the group with 0.2% Attapulgite Clay-grafted acrylic acid–acrylamide hydrogel (ACH) added was larger compared to the group with 0% ACH added. Under mild alkali stress (AS1), adding 0.2% ACH also increased the leaf area of spinach. In general, the addition of ACH can effectively alleviate the inhibitory effect of salt–alkali stress on spinach growth and improve its growth status.

### 2.4. Effect of ACH Addition on Chlorophyll Content of Spinach

**Figure 5:**
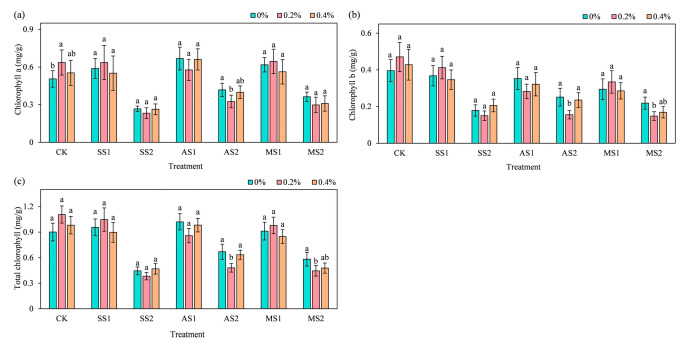
Effect of addition of ACH and SS, AS, and MS on chlorophyll content of spinach. In the figure, lowercase letters indicate statistically significant differences between different ACH addition groups (*p* < 0.05). (**a**) Chlorophyll a; (**b**) Chlorophyll b; (**c**) Total chlorophyll.

As shown in Figure 5a, in the CK treatment group, the addition of 0.2% ACH significantly increased chlorophyll a content by 25.95% (*p* < 0.05) compared to the addition of 0% ACH, while the addition of 0.4% ACH increased chlorophyll content by 9.52%. Overall, after the application of stress, the addition of ACH had no significant effect on chlorophyll a, chlorophyll b, or total chlorophyll content.

### 2.5. Effect of ACH Addition on the Osmoregulatory Substances in Spinach

#### 2.5.1. Proline

**Figure 6:**
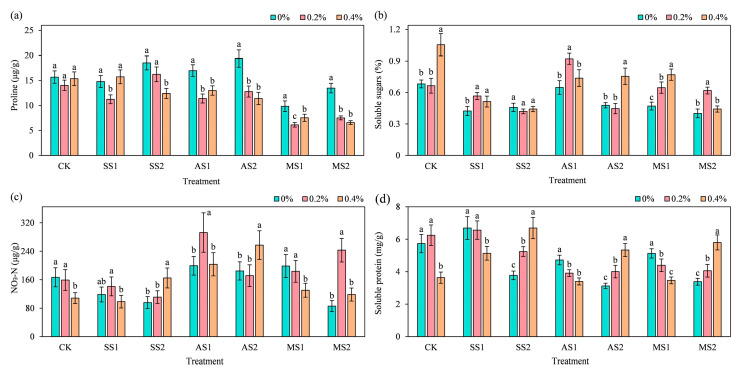
Effect of addition of ACH and SS, AS, and MS on osmoregulatory substances in spinach. In the figure, lowercase letters indicate statistically significant differences between different ACH addition groups (*p* < 0.05). (**a**) Proline; (**b**) Soluble sugars; (**c**) NO_3_-N; (**d**) Soluble protein.

As shown in Figure 6a, overall, the addition of ACH significantly reduced the proline content of spinach after stress application. Under mild stress conditions, the MS1 treatment group with 0.2% ACH showed the most significant decrease in proline content compared to the group with 0% ACH, at 37.83% (*p* < 0.05). Under severe stress conditions in the MS2 treatment group, the addition of 0.4% ACH resulted in the most significant decrease in spinach proline content compared to the group without ACH, with a reduction of 51.25% (*p* < 0.05). Regardless of the stress severity (mild or severe), the application of ACH consistently led to a significant reduction in the proline content of spinach following stress exposure (*p* < 0.05), evidencing that ACH exerts a positive effect on the physiological regulation of stress resistance in spinach.

#### 2.5.2. Soluble Sugars

As shown in Figure 6b, the CK treatment group showed a significant increase of 54.6% in soluble sugar content in spinach when 0.4% ACH was added (*p* < 0.05). In the AS1 treatment group (mild alkaline stress), adding 0.2% ACH compared to adding 0% ACH significantly increased the soluble sugar content by 42.15% (*p* < 0.05). In the AS2 treatment group (severe alkaline stress), adding 0.4% ACH compared to adding 0% ACH significantly increased the soluble sugar content by 57.83% (*p* < 0.05). In the MS1 treatment group (under mild mixed salt–alkali stress), the addition of 0.4% ACH compared to 0% ACH resulted in a significant increase of 63.54% in soluble sugar content (*p* < 0.05). In the MS2 treatment group (under severe mixed salt–alkali stress), the addition of 0.2% ACH compared to 0% ACH resulted in a significant increase of 54.43% in soluble sugar content (*p* < 0.05). Soluble sugars, as key osmotic adjustment and energy storage substances in plants, enhance plant cells’ osmotic regulation capacity and tolerance to adverse environments (e.g., alkaline and mixed salt–alkali stresses) when their content increases; ACH may thus help spinach better adapt to stressful environments by boosting soluble sugar levels.

#### 2.5.3. Nitrate Nitrogen (NO_3_^−^-N)

As shown in Figure 6c, in the CK treatment group, as ACH content increased, the nitrate-nitrogen content in spinach showed a decreasing trend. When 0.4% ACH was added, the nitrate-nitrogen content decreased significantly by 34.98% (*p* < 0.05). Under SS1 (mild salt stress) treatment, adding 0.2% ACH increased the nitrate-nitrogen content of spinach by 19.13%. As the stress intensity increased, under SS2 treatment, adding 0.4% ACH significantly increased the nitrate-nitrogen content of spinach by 72.37% (*p* < 0.05). Among alkaline stress treatments (AS1, AS2), in the AS1 treatment (mild alkaline stress), the addition of 0.2% ACH resulted in the most significant increase in nitrate-nitrogen content, at 46.88% (*p* < 0.05). Under mild mixed salt–alkali stress in the MS1 treatment group, adding 0.4% ACH significantly reduced the nitrate-nitrogen content of spinach by 34.3% (*p* < 0.05). In the MS2 treatment group under severe mixed salt–alkali stress, the addition of 0.2% ACH significantly increased the nitrate-nitrogen content of spinach by 183.26% (*p* < 0.05).

#### 2.5.4. Soluble Protein

As shown in Figure 6d, in the CK treatment group, when the ACH addition rate was 0.4%, the soluble protein content of spinach decreased significantly by 36.66% (*p* < 0.05). Under low-stress conditions (SS1, AS1, MS1), adding 0.4% ACH significantly reduced the soluble protein content of spinach, with the MS1 group (mild saline–alkali mixed stress) showing the most significant decrease of 100% (*p* < 0.05). As stress intensity increased, adding 0.4% ACH under high-stress conditions (SS2, AS2, MS2) significantly increased spinach soluble protein content. Among treatments, the SS2 treatment group (severe salt stress) showed a significant increase of 77.26% in soluble protein content (*p* < 0.05).

### 2.6. Effect of ACH Addition on Antioxidant Enzymes and Membrane Damage Indices in Spinach

#### 2.6.1. Superoxide Dismutase

Overall, without the addition of ACH, under mild stress conditions (SS1, AS1, MS1), the superoxide dismutase (SOD) activity of spinach increased compared to the CK treatment, while under severe stress conditions (SS2, AS2, MS2), SOD activity decreased compared to the CK treatment. In the CK treatment, adding 0.4% ACH significantly reduced enzyme activity by 40.06% compared to adding 0% hydrogel (*p* < 0.05). Under low-stress conditions (SS1, AS1, MS1), adding 0.4% ACH significantly reduced SOD activity (*p* < 0.05). Under high-stress conditions (SS2, AS2, MS2), the addition of 0.4% ACH significantly increased enzyme activity (*p* < 0.05). Specifically, under SS2 treatment (severe salt stress), the addition of 0.4% ACH significantly increased spinach SOD activity by 65.43% compared to 0% ACH (*p* < 0.05).

#### 2.6.2. Peroxidase

**Figure 7:**
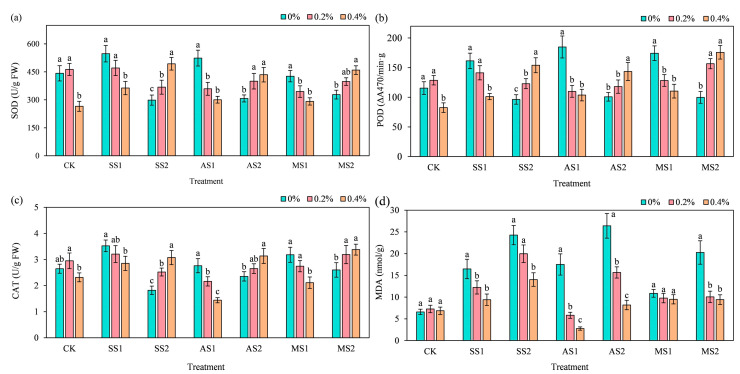
Effect of addition of ACH and SS, AS, and MS on antioxidant enzymes and membrane damage indices in spinach. In the figure, lowercase letters indicate statistically significant differences between different ACH addition groups (*p* < 0.05). (**a**) SOD; (**b**) POD; (**c**) CAT; (**d**) MDA.

As shown in Figure 7b, in the CK treatment group, the addition of 0.4% ACH significantly reduced the peroxidase (POD) activity of spinach by 28.46% (*p* < 0.05). Under low-stress conditions (SS1, AS1, MS1), the addition of 0.4% ACH in the AS1 (mild alkaline stress) treatment group resulted in the most significant decrease in spinach POD activity, at 43.92% (*p* < 0.05). In the high-stress treatment groups (SS2, AS2, MS2), the addition of ACH increased spinach POD activity. Among treatments, the addition of 0.4% ACH in the MS2 treatment group (severe salt–alkali mixed stress) resulted in the most significant increase in spinach POD activity compared to the addition of 0% ACH, at 76.21% (*p* < 0.05).

#### 2.6.3. Catalase

Under low-stress conditions (SS1, AS1, MS1), the addition of 0.4% ACH in the MS1 treatment group (mild mixed salt–alkali stress) resulted in the most significant decrease in spinach Catalase (CAT) activity, at 33.66% (*p* < 0.05). In the high-stress treatment groups (SS2, AS2, MS2), the addition of ACH increased spinach CAT activity. Among treatments, the addition of 0.4% ACH in the SS2 treatment group (severe salt stress) resulted in the most significant increase in spinach CAT activity compared to the addition of 0% ACH, with a 68.99% increase (*p* < 0.05).

#### 2.6.4. Malondialdehyde

As shown in Figure 7d, the addition of ACH had no significant effect on the malondialdehyde (MDA) content of spinach in the CK treatment group. Compared with the CK treatment, the MDA content of spinach increased to varying degrees after stress application, but with the addition of ACH, the MDA content showed a decreasing trend. Under low-stress conditions (SS1, AS1, MS1), the AS1 treatment group (mild alkaline stress) showed the most significant decrease in MDA content in spinach when 0.4% ACH was added, with a reduction of 84.14% (*p* < 0.05). Under high-stress conditions (SS2, AS2, MS2), the AS2 treatment group (severe alkaline stress) showed the most significant decrease in MDA content in spinach, with a reduction of 68.98% (*p* < 0.05) when 0.4% ACH was added. Regarding the regulation of spinach malondialdehyde (MDA) content by ACH, ACH had no significant effect in the non-stressed CK group, whereas, in various stress environments, ACH could significantly reduce MDA content, thereby alleviating stress-induced membrane damage. Notably, 0.4% ACH exhibited the most prominent mitigating effect in the alkaline stress groups (AS1, AS2).

### 2.7. Effect of ACH on the Distribution of K^+^ and Na^+^ in Spinach

#### 2.7.1. K^+^ and Na^+^ Content in Spinach Leaves

**Figure 8:**
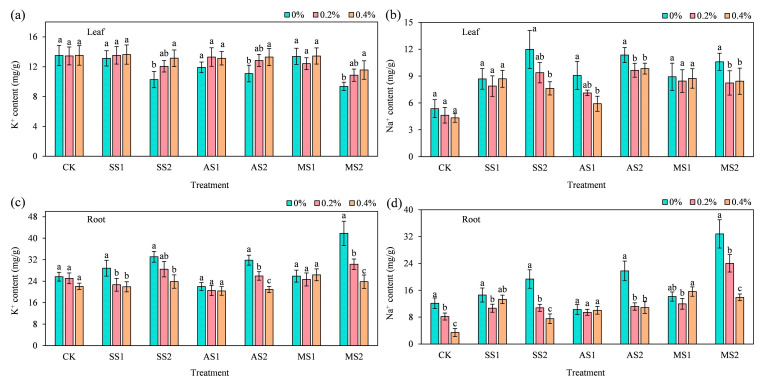
Effect of addition of ACH and SS, AS, and MS on K^+^ and Na^+^ content in spinach. In the figure, lowercase letters indicate statistically significant differences between different ACH addition groups (*p* < 0.05). (**a**) Leaf K^+^; (**b**) Leaf Na^+^; (**c**) Root K^+^; (**d**) Root Na^+^.

As shown in Figure 8a, overall, under low-stress conditions (SS1, AS1, MS1), the addition of ACH did not significantly alter the K^+^ content in spinach leaves; however, under high-stress conditions (SS2, AS2, MS2), the addition of 0.4% ACH significantly increased the K^+^ content in the leaves. Among treatments, the enhancement effect was most pronounced under severe salt stress treatment (SS2), with leaf K^+^ content significantly increased by 27.82% compared to the 0% ACH treatment (*p* < 0.05). As shown in Figure 8b, compared to the CK treatment, Na^+^ content in leaves increased to varying degrees after stress application. In the CK treatment group, the addition of ACH had no significant effect on Na^+^ content in the leaves. Under SS2, AS2, and MS2 treatments (severe salt–alkali stress), the addition of 0.4% ACH significantly reduced Na^+^ content in spinach leaves by 36.4%, 13.34%, and 20.43%, respectively (*p* < 0.05). Under the AS1 treatment (mild alkaline stress), the addition of 0.4% ACH significantly reduced the Na^+^ content in spinach leaves by 34.94% (*p* < 0.05). ACH increased spinach leaf K^+^ levels significantly only under high stress and was notably most effective in the SS2 group (severe salt stress), while it significantly reduced Na^+^ levels in both the AS1 group (mild alkaline stress) and all high-stress groups.

#### 2.7.2. K^+^ and Na^+^ Content in Spinach Roots

As shown in Figure 8c, in the CK treatment group, the addition of ACH had no significant effect on K^+^ content in spinach roots. In the mild salt stress treatment group (SS1), the addition of 0.4% ACH significantly reduced K^+^ content in spinach roots by 24.06% (*p* < 0.05). Under severe stress conditions (SS2, AS2, MS2), the addition of 0.4% ACH in the MS2 treatment group (severe saline–alkali stress) resulted in the most significant decrease in K^+^ content in the roots, at 43.03% (*p* < 0.05). This indicates that the addition of ACH can alleviate the accumulation of K^+^ in the root system. Compared to the CK treatment, Na^+^ content in spinach roots increased to varying degrees after stress application. However, after adding ACH, Na^+^ content in spinach roots showed a decreasing trend. Under low-stress treatments (SS1, AS1, MS1), the addition of 0.2% ACH in the SS1 treatment (mild salt stress) resulted in the most significant decrease in root Na^+^ content, at 26.95% (*p* < 0.05). Under high-stress treatments (SS2, AS2, MS2), the addition of 0.4% ACH resulted in the most significant decrease in Na^+^ content in the roots, reaching 61.12% (*p* < 0.05) under the SS2 treatment (severe salt stress); this combination was the optimal one for reducing root Na^+^ content among all treatments.

### 2.8. Correlation Analysis

**Figure 9:**
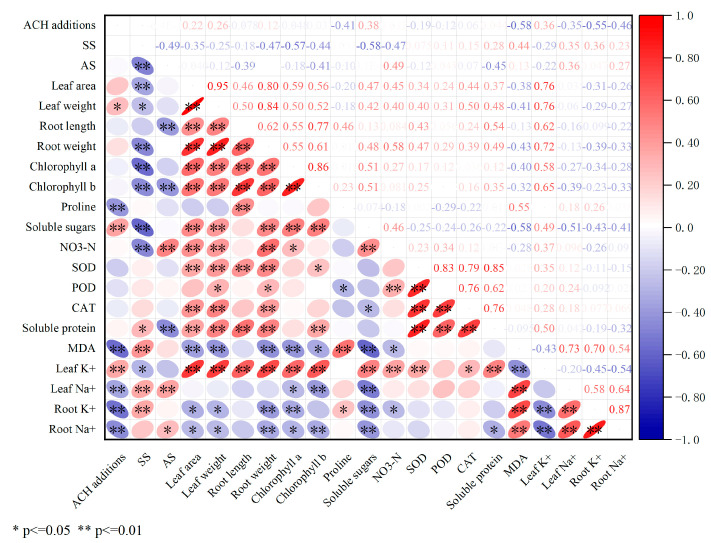
The relationship between spinach growth and physiological indices (note: SS in the figure refers to salt stress; AS refers to alkali stress).

As shown in Figure 9, the addition of ACH is significantly positively correlated with leaf weight (r = 0.26, *p* < 0.05), indicating that the addition of ACH can effectively increase spinach yield. ACH addition is significantly negatively correlated with proline (r = −0.41, *p* < 0.01), malondialdehyde (MDA) (r = −0.58, *p* < 0.01), root K^+^ (r = −0.55, *p* < 0.01), and root Na^+^ (r = −0.46, *p* < 0.01), indicating that the addition of ACH can inhibit the excessive accumulation of proline, alleviate membrane lipid peroxidation to protect cell membranes, and regulate root ion absorption to maintain “potassium–sodium balance,” thereby alleviating salt stress from the ion homeostasis level. Under salt stress (SS), there was a significant negative correlation with chlorophyll a (r = −0.57, *p* < 0.01) and soluble sugars (r = −0.58, *p* < 0.01), indicating that salt stress disrupts chlorophyll synthesis, inhibits the photosynthetic system, and interferes with soluble sugar metabolism. ACH can weaken this negative correlation, alleviate the inhibition of photosynthesis and carbon metabolism caused by salt damage, and help plants maintain osmotic homeostasis.

Under alkaline stress (AS), there was a significant negative correlation between chlorophyll b (r = −0.41, *p* < 0.01) and soluble protein (r = −0.45, *p* < 0.01), indicating that alkaline stress inhibits chlorophyll b synthesis and soluble protein accumulation, thereby reducing photosynthetic efficiency and stress tolerance. ACH reduces the degree of negative correlation, alleviates alkaline stress impact, and enhances alkaline tolerance. Chlorophyll a and chlorophyll b (r = 0.86, *p* < 0.01), leaf area and leaf weight (r = 0.95, *p* < 0.01), leaf area and root weight (r = 0.80, *p* < 0.05), and leaf area and leaf potassium (r = 0.86, *p* < 0.01) showed significant positive correlations. An increase in leaf area promotes biomass accumulation and synergistically enhances leaf potassium content. SOD was significantly positively correlated with soluble protein (r = 0.85, *p* < 0.01), SOD with CAT (r = 0.79, *p* < 0.01), and SOD with POD (r = 0.83, *p* < 0.01).

### 2.9. Principal Component Analysis (PCA) and Cluster Analysis

**Figure 10:**
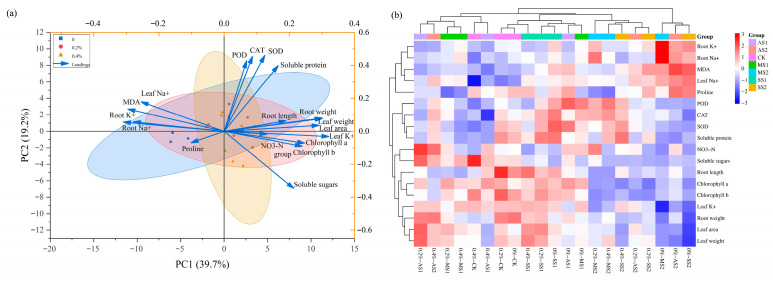
Principal component analysis (grouped by different ACH addition levels) (**a**); cluster analysis (**b**). (Note: In the grouping, 0%, 0.2%, and 0.4% are ACH addition levels; SS1 refers to mild saline–alkali stress; SS2 refers to severe salt stress; AS1 refers to mild alkali stress; AS2 refers to severe alkali stress; MS1 refers to mild mixed saline–alkali stress; MS2 refers to severe mixed saline–alkali stress.)

As shown in Figure 10a, the PCA diagram clearly illustrates the regulatory effect of ACH addition on physiological growth indicators of spinach. It explains how ACH influences the synergistic relationship between stress resistance and growth in spinach from the perspectives of indicator correlations, treatment effects, and stress response mechanisms. The 0% ACH sample points (blue squares) are shifted to the left, indicating significant negative effects from leaf Na^+^ and MDA, resulting in weaker growth (e.g., root weight, leaf weight) and photosynthetic (chlorophyll) indicators. This suggests that without ACH addition, spinach is more susceptible to salt–alkali stress damage. The 0.2% ACH sample points (red dots) are shifted to the right, showing good synergy among growth, photosynthesis, and antioxidant enzyme indicators, indicating that 0.2% hydrogel can effectively alleviate salt–alkali stress while regulating ion balance, promoting a balance between spinach growth and stress resistance. The 0.4% ACH sample point (yellow triangle) shows a high correlation with proline osmotic regulation substances, indicating that spinach needs to accumulate proline to cope with saline–alkali stress at this stage. POD, CAT, and SOD (antioxidant enzymes) show similar arrow directions with growth indicators such as leaf weight, area, chlorophyll (a, b, and total), root weight, and length, indicating that a strong antioxidant system promotes spinach growth and photosynthesis; soluble proteins are also positively correlated with these indicators, playing a positive role in physiological regulation. Leaf Na^+^, MDA (a marker of membrane damage), and Na^+^ in roots and leaves show opposite arrow directions compared to growth and antioxidant indicators, indicating that high Na^+^ accumulation triggers membrane lipid peroxidation, inhibiting photosynthesis and growth, making it a key factor in salt-stress-induced inhibition of spinach growth. When spinach responds to salt stress, Na^+^ accumulation increases MDA levels and inhibits growth, while antioxidant enzymes and proline mitigate damage through negative feedback regulation, explaining the synergistic mechanism of interrelated physiological indicators under stress conditions.

### 2.10. Cluster Analysis

Figure 10b shows a cluster analysis of spinach under saline–alkali stress based on multiple physiological and biochemical indicators. Leaf area, leaf weight, root weight, and leaf K^+^ indicators are grouped into one category, while growth indicators such as leaf area and root length, photosynthetic and material synthesis indicators such as chlorophyll and soluble sugars, and nutrient indicators such as nitrate nitrogen are grouped into another category. These indicators reflect the plant’s basic growth, material production, and nutrient utilization, serving as foundational physiological indicators for survival under adverse conditions. They are the first to exhibit changes such as growth inhibition and impaired material synthesis when subjected to stress. Antioxidant enzymes such as SOD, CAT, and POD, osmotic regulation substances such as proline, and membrane damage indicators such as MDA are grouped together. These correspond to oxidative stress, osmotic regulation, and membrane protection triggered by stress, forming the plant’s “stress defense module.” Stress such as salinity activates these mechanisms, which counteract damage by scavenging reactive oxygen species, regulating osmotic pressure, and repairing membrane damage. Leaf Na^+^ and root Na^+^, among others, are related to the distribution of sodium and potassium ions in roots and leaves, reflecting physiological responses to ion absorption, transport, and distribution under saline–alkali stress. This group of indicators reflects the plant’s maintenance of intracellular ion homeostasis, which is crucial for basic functions such as cellular osmotic pressure and enzyme activity under stress conditions. Cluster analysis further corroborates the results of the PCA plot. Combining the PCA plot and cluster analysis results, it is evident that ACH can alleviate the inhibitory effects of saline–alkali stress on spinach growth and photosynthesis through multiple pathways, including regulating ion balance, activating the antioxidant system, and modulating osmotic substances.

### 2.11. Path Analysis

**Figure 11:**
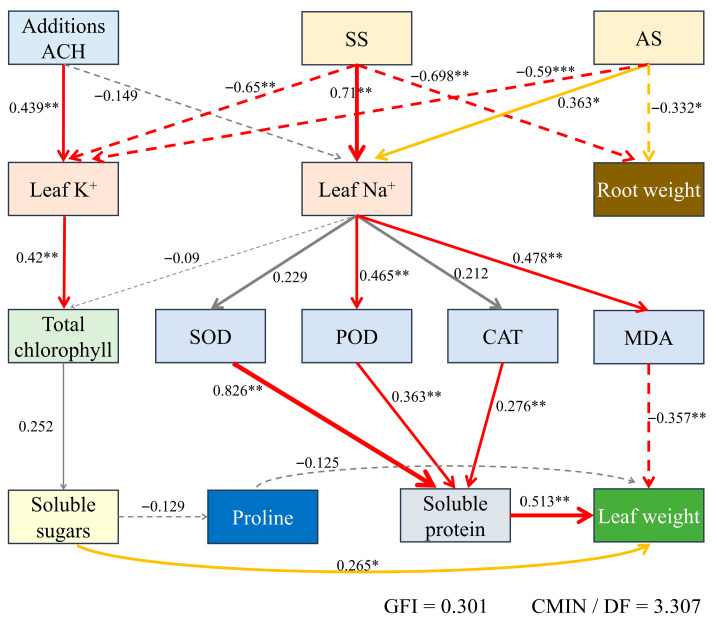
Path analysis. (In the figure, SS denotes salt stress, AS denotes alkaline stress, and ACH denotes Attapulgite Clay-g-(AA-co-AAm) Hydrogel. Red lines indicate *p* < 0.05, yellow lines indicate *p* < 0.1, and gray lines indicate no significance. Solid lines indicate positive correlation, dashed lines indicate negative correlation, and thicker lines indicate stronger correlation). *** denotes *p* < 0.001,** denotes *p* < 0.01, while * denotes *p* < 0.05.

This path analysis model focuses on the effects of salt stress (SS), alkaline stress (AS), and the addition of Attapulgite Clay-g-(AA-co-AAm) Hydrogel (ACH) on plant physiology, revealing response mechanisms ranging from ion balance to antioxidant systems, photosynthetic performance, and biomass accumulation. SS and AS significantly disrupt leaf ion balance. SS causes a significant accumulation of Na^+^ in leaves (0.71, *p* < 0.01) and inhibits K^+^ absorption (−0.65, *p* < 0.01). AS has a slightly weaker effect on Na^+^ (0.363, *p* < 0.01) but significantly reduces K^+^ content (−0.59, *p* < 0.01). In contrast, ACH addition had no significant effect on leaf Na^+^ but promoted K^+^ absorption (0.439, *p* < 0.05), thereby alleviating ion imbalance under stress to some extent. Increased Na^+^ in leaves leads to membrane lipid peroxidation, elevated MDA content (0.478, *p* < 0.05), and increased peroxidase activity (0.465, *p* < 0.05), which are defensive mechanisms of plants in response to stress. Increased K^+^ has a positive effect on total chlorophyll content (0.42, *p* < 0.05), ensuring photosynthesis. SOD, POD, and CAT promote soluble protein synthesis. Soluble sugars promote leaf weight increase (*p* < 0.01), while MDA inhibits leaf growth (−0.357, *p* < 0.05). Conversely, increased soluble protein content significantly promotes leaf weight increase (0.513, *p* < 0.05). SS and AS both significantly inhibit root growth, with root weight significantly reduced under SS conditions (−0.698, *p* < 0.01) and AS having a similar effect (−0.332, *p* < 0.05), as plants prioritize energy use to respond to stress. SS and AS disrupt leaf ion balance, affecting the plant’s antioxidant system, photosynthetic performance, and biomass accumulation. ACH addition helps improve leaf K^+^ absorption and has a positive effect in alleviating stress impacts (see Figure 11).

## 3. Discussion

### 3.1. Surface Characteristics and Properties of ACH

The ACH prepared in this study exhibits a loose, porous structure. This aligns with the findings of Xie et al. [15] regarding Attapulgite Clay–polymer composite hydrogels, where the introduction of inorganic clay disrupts the compact packing of polymer chains, forming more interconnected pores to enhance specific surface area and adsorption performance. Archibong et al. [16] also observed similar patterns of pore structure changes in the montmorillonite–polymer system.

Infrared spectroscopy shows that ACH retains organic functional groups (amide groups, carboxyl groups) and inorganic Si-O bonds, and the functional group vibration peaks vary with the addition of Attapulgite Clay, which is consistent with the findings of Ogawa, Ruiz-Fresneda et al. [17,18] in montmorillonite–acrylamide hydrogels. Wang et al. [19] also validated this pattern in their study on sepiolite–polyacrylic acid hydrogels, providing a structural basis for ACH’s mitigation of saline–alkali stress. The ACH prepared in this study exhibits a loose, porous structure that becomes more refined with increasing Attapulgite Clay content, consistent with previous studies on clay–polymer composite hydrogels [18]. This confirms the critical role of inorganic clay in optimizing hydrogel pore structure and enhancing adsorption performance.

### 3.2. Mechanisms of ACH Effect on Physiological Growth Indices of Spinach Tolerant to Salinity Stress

The promotional effect of ACH on spinach growth was most significant under severe salt stress (SS2) (0.2% ACH increased leaf area by 91% and leaf weight by 95.69%), consistent with the findings of Ahmed et al. [20], who found that clay-based hydrogels can increase leaf area in tomatoes under salt stress. It is speculated that ACH promotes nutrient absorption by improving the rhizosphere microenvironment. ACH may possess hydrophilic groups or porous structures similar to those of hydrogels. On the one hand, it can maintain suitable soil moisture in the rhizosphere through strong water absorption and retention capabilities [21], preventing water uptake difficulties in roots caused by soil drought under severe salt stress, thereby providing the basic moisture conditions for nutrient dissolution and root absorption; on the other hand, it may adsorb excess salt ions (such as Na^+^) in the rhizosphere soil through surface charge, thereby reducing the concentration of salt ions in the rhizosphere, alleviating damage to root cell membranes caused by high salt levels and maintaining the root system’s ability to absorb essential nutrients, such as nitrogen, phosphorus, and potassium [22]. In mild alkaline stress conditions, the alleviating effect of low-concentration ACH (46.3% increase in leaf area) aligns with the findings of Liu et al. [23], indicating that the stress-alleviating effects of such functional regulatory substances are not continuously enhanced with increasing concentration but exhibit concentration-dependent specificity, meaning their efficacy varies at different concentrations. This phenomenon is also consistent with the conclusion drawn by Rychter P et al. [24] regarding the regulation of rhizosphere pH by low-concentration hydrogels in cucumbers. Under severe salt stress, high concentrations of Na^+^ lead to increased soil solution osmotic pressure, making it difficult for plant roots to absorb water, while the porous structure of ACH can alleviate water stress through its strong water-holding capacity [5,25,26]. Additionally, the adsorption of Na^+^ by its surface functional groups [26] reduces the concentration of free Na^+^ in the rhizosphere environment, mitigating ionic toxicity and thereby more significantly promoting leaf growth and biomass accumulation. Under mild alkaline stress, elevated soil pH is the primary limiting factor. Low concentrations of ACH can regulate soil buffering capacity [27], reduce pH fluctuations in the rhizosphere, and provide a more suitable acid–base environment for the root system, thereby promoting nutrient absorption and leaf expansion. This is consistent with the mechanism proposed by Chakraborty et al. [28], where hydrogels alleviate stress by improving the rhizosphere chemical environment.

At the physiological index level, the regulatory effect of ACH on osmotic adjustment substances and antioxidant systems further reveals the intrinsic mechanism by which it alleviates saline–alkali stress. In terms of osmotic adjustment substances, ACH can significantly reduce the proline content in spinach. Under severe mixed stress (MS2), treatment with 0.4% ACH reduced the proline content by 51.25%, which is consistent with the conclusion proposed by Kant, Sayed et al. [10,29] that “hydrogels can reduce proline accumulation in plants under salt stress”. As an important osmotic adjustment substance for plants to cope with adverse environments, excessively high proline content usually indicates that plants are in a state of extreme stress. By reducing the proline content, ACH indirectly shows that it effectively alleviates the damage to spinach from saline–alkali stress. Meanwhile, ACH can significantly increase the soluble sugar content. Under mild mixed saline–alkali stress (MS1), treatment with 0.4% ACH increased the soluble sugar content by 63.54%. This result provides strong support for the mechanism proposed by Fang, Li et al. [14,30] that “hydrogels maintain cellular osmotic pressure through soluble sugars”.

### 3.3. The Regulatory Mechanism of ACH on Na^+^ and K^+^ in Spinach

From the perspective of Na^+^ regulation, ACH significantly reduces Na^+^ accumulation in spinach roots and leaves: e.g., under severe salt stress (SS2), 0.4% ACH decreased root and leaf Na^+^ contents by 61.12% and 36.4%, respectively. This effect is linked to ACH’s unique microstructure and chemical composition—its 5% montmorillonite forms a loose porous network, markedly enhancing specific surface area and providing abundant active sites for ion adsorption; additionally, ACH’s surface carboxyl (-COOH) and amide (-CONH_2_) groups exhibit strong electronegativity, which binds Na^+^ via electrostatic attraction. This effectively lowers free Na^+^ concentration in rhizosphere soil solution and weakens roots’ passive absorption of Na^+^ [9,31].

ACH enhances plant stress resistance by regulating K^+^ levels, promoting leaf K^+^ accumulation: e.g., under severe salt stress (SS2), 0.4% ACH significantly increased spinach leaf K^+^ content by 27.82%, key to maintaining leaf physiological metabolism and growth. K^+^, as an indispensable cation within plant cells, participates in numerous important physiological processes such as enzyme activity regulation, osmotic potential maintenance, and stomatal movement control, playing a crucial role in maintaining normal physiological metabolism and growth development of leaves. The mechanism by which ACH promotes K^+^ transport to the aboveground parts may involve multiple levels. First, salt stress deteriorates the rhizosphere microenvironment and weakens root system vitality, while ACH can optimize soil structure, regulate pH, and enhance root system vitality, indirectly improving leaf K^+^ absorption efficiency, thereby alleviating physiological disorders caused by K^+^ deficiency and reducing stress damage [32,33]. Second, saline–alkali stress disrupts the function of K^+^ transport proteins such as AKT1 and HAK5 in root cell membranes. ACH alleviates saline–alkali stress, reduces the excessive transport of Na^+^ by transport proteins, and ensures that K^+^ can still be absorbed and transported to leaves under stress conditions, maintaining plant ion balance [34,35].

Correlation analysis further validated the synergistic regulatory effect of ACH on the Na^+^-and-K^+^ ion balance in spinach. The addition of ACH showed a significant negative correlation with Na^+^ content in spinach roots and leaves (r = −0.46, *p* < 0.01), which is highly consistent with previous studies indicating that clay-based hydrogels can reduce Na^+^ accumulation in plants [36,37]. Additionally, the amount of ACH added showed a significant positive correlation with leaf biomass (r = 0.26, *p* < 0.05), strongly supporting the view that optimizing ion balance (reducing Na^+^ toxicity and promoting reasonable K^+^ distribution) can effectively promote aboveground plant growth [22,38]. This synergistic regulatory mechanism of “reducing Na^+^ accumulation and promoting K^+^ transport” ultimately maintains a high K^+^/Na^+^ ratio within spinach cells. This is of critical importance for stabilizing cell membrane structure, maintaining enzyme activity, regulating cellular osmotic pressure balance, and enhancing spinach’s tolerance to saline–alkali stress.

## 4. Materials and Methods

### 4.1. Experimental Material

All reagents used in this experiment were purchased from Sinopharm Chemical Reagent Co., Ltd. (Shanghai, China), with analytical purity. The soil used in the pot experiment, specifically typical loessial soil, was collected from the field. Its main physical and chemical properties are as follows: soil organic carbon content is 7.4 g/kg, total nitrogen content is 0.93 g/kg, pH value is 8.79, and bulk density is 1.2 g/cm^3^. The spinach variety used in this study is the traditional large-leaf spinach.

### 4.2. Preparation of Attapulgite Clay-P (AA-co-AAm) Hydrogels (ACHs)

**Figure 12:**
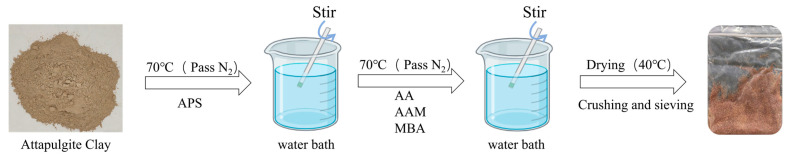
The synthesis process of Attapulgite Clay-g-(AA-co-AAm) Hydrogel (ACH).

In this study, attapulgite from Gannan, Jiangxi, China, was used to prepare 500 g of (AA-co-AAm)/AC Hydrogel. The synthesis process is as follows: Fully crushed Aconitum (AC) was mixed with 300 mL of deionized water (60% of total mass) to form a suspension, which was transferred to a three-necked flask, purged with N_2_ for 30 min, and heated to 70 °C in a water bath. Then, APS was added and stirred for 30 min, followed by AAm (stirred 30 min), AA (stirred 30 min), cross-linking agent, and remaining deionized water (29.8% of total mass) with 30 min stirring. The mixture was heated in the water bath until gel formation. The gel was washed 3 times with ethanol (to remove unreacted monomers) and 3 times with ultrapure water (to remove residual ethanol), then dried at 40 °C, crushed, and sieved through a 0.5 mm sieve. The synthesis process of Attapulgite Clay-g-(AA-co-AAm) Hydrogel (ACH) is illustrated in Figure 12 below.

In this experiment, attapulgite-grafted copolymerized acrylic acid–acrylamide hydrogels (ACHs) were prepared with attapulgite addition levels set at 2.5% (low addition level, ACH-1) and 5% (high addition level, ACH-2) in two gradients. The ACH grafting rate is calculated by the volume comparison method. Specifically, the total volume of ACH obtained during the preparation process is compared and calculated with the total volume of ACH after drying, and the ACH grafting rate is derived based on this. Through determination and calculation, the grafting rate of ACH-1 attapulgite is 87.7%, and that of ACH-2 attapulgite is 70.4%. The results showed that the surface roughness of the ACH with a 5% (high) addition rate was significantly higher than that of the ACH with a 2.5% (low) addition rate, exhibiting a more porous and loose microstructure.

(1) Grafting rate = ((Attapulgite Clay addition amount + APS + AA + AAM + MBA) − Gel after drying and pulverization)/Attapulgite Clay addition amount

### 4.3. ACH Characterization Tests

The samples were tested using a Fourier Transform Infrared Spectrometer (Thermo Fisher Nicolet iS50, Thermo Fisher Scientific, Waltham, MA, USA) to obtain infrared spectrograms in the wavelength range of 400–4000 cm^−1^. The samples were then subjected to a rapid pressing process at 10–15 MPa. During the test, the powder samples were rapidly pressed under a pressure of 10–15 MPa. The surface morphology of the samples was observed with the aid of a scanning electron microscope (SEM, JEOL S-3400N, Hitachi, Tokyo, Japan), and the samples were sprayed with gold before observation. The SEM has a working distance of 53 mm and an operating voltage of 10 kV.

### 4.4. Testing of Salinity Tolerance of Spinach by ACH Addition

This experiment selected ACH-2 (the addition amount of attapulgite accounts for 5% of the total mass), aiming to utilize its richer pore structure and higher surface roughness to enhance the mitigation effect on saline–alkali stress. In the experiment, ACH was incorporated into the soil at a rate based on the mass of potting soil during the initial stage of soil preparation; whereas the saline–alkali stress treatments are set as follows: All stress levels were calculated based on the mass of potting soil (for instance, an addition of 0.2% refers to adding the corresponding solution at 0.2% of the potting soil mass). Salt stress (SS) was induced using NaCl solution, with an addition rate of 0.2% for mild salt stress and 0.4% for severe salt stress; alkali stress (AS) was induced using NaHCO_3_ solution, with an addition rate of 0.2% for mild alkali stress and 0.4% for severe alkali stress; and salt–alkali mixed stress (MS) was induced using a solution of NaCl and NaHCO_3_ mixed in a 1:1 ratio, with an addition rate of 0.2% for mild salt–alkali mixed stress and 0.4% for severe salt–alkali mixed stress. And all saline–alkali stresses were imposed when the spinach plants reached 15 days old. In the design of this experiment, each treatment group was set with 3 replicates, and the spatial arrangement was conducted through a randomized block design to ensure the balance of experimental conditions. The pot experiment lasted for 45 days and was conducted in the greenhouse of the College of Resources and Environment, Gansu Agricultural University, from May to June 2025. For watering management, irrigation was performed daily between 17:00 and 18:00, and the irrigation amount was controlled at 75% of the field capacity (see Table 1).

### 4.5. Measurement of Growth Indices

#### 4.5.1. Biomass Determination

After the potted plant experiment, the entire spinach plant was removed from the pot, and the aboveground and underground parts were separated. The leaf area of the aboveground part was determined using the paper weight method, and the root length was measured with a ruler. A precision balance was used to weigh the aboveground and underground parts separately, and the fresh weights of the spinach leaves and roots were recorded. Subsequently, the samples were blanched at 90 °C for 30 min, then transferred to a 60 °C oven and dried to constant weight. After weighing the dry weight of the aboveground and underground parts, the total biomass and root-to-shoot ratio (dry weight of the underground part/dry weight of the aboveground part) were calculated.

(2) Total biomass = aboveground dry weight + belowground dry weight

(3) Root–shoot ratio = dry weight below ground/dry weight above ground

#### 4.5.2. Leaf Area Measurement (Paper Weight Method)

This method is based on the characteristic that the unit area weight of homogeneous cardboard remains constant. The specific steps are as follows: First, take a piece of uniformly shaped homogeneous cardboard (such as a square or rectangle), weigh it using a precision balance, and calculate the unit area weight of the cardboard (unit: g/cm^2^) based on the measured length and width. Lay the entire leaf flat on the paperboard, trace the outline precisely along the edge of the leaf, and then cut out the corresponding section of paperboard along the outline and weigh it. The leaf area calculation formula is as follows:

(4) Leaf area = Weight of cut cardboard/Unit weight of cardboard

### 4.6. Measurement of Physiological Indices

(1) Chlorophyll Determination:

The determination of chlorophyll content involves weighing 0.2–0.5 g of chopped leaf samples, extracting them with 95% ethanol, and measuring the color at wavelengths of 645 nm and 663 nm using a spectrophotometer.

(2) Determination of Osmotic Adjustment Substances:

Proline determination involves weighing 0.5–1 g of plant sample, extracting it with 3% sulfosalicylic acid in a boiling water bath, and measuring the absorbance at 520 nm using a spectrophotometer.

The soluble sugar content is determined by weighing 0.1–0.5 g of plant sample, extracting it with distilled water in a boiling water bath, and measuring the absorbance at 620 nm using a spectrophotometer. The soluble sugar content is then determined using a glucose standard curve.

For nitrate-nitrogen determination, approximately 0.5 g of plant sample is weighed, extracted with distilled water under boiling water bath conditions, and measured using a spectrophotometer at a wavelength of 410 nm.

Soluble protein is extracted using phosphate-buffered solution, with the extraction process including grinding and centrifugation steps. The absorbance is measured using a spectrophotometer at a wavelength of 595 nm.

(3) Determination of Antioxidant Enzymes and Malondialdehyde (MDA) Content:

For SOD determination, approximately 0.5 g of plant sample is weighed, ground into a homogeneous paste with pre-chilled phosphate-buffered solution (pH 7.8), and then centrifuged at 10,000 rpm and 4 °C for 15 min. The resulting supernatant serves as the crude extract for SOD, POD, CAT, and MDA. SOD measurement was performed using the NBT photoreduction method, with absorbance measured at a wavelength of 560 nm using a spectrophotometer.

POD measurement was performed using the guaiacol method, with absorbance measured at a wavelength of 470 nm using a spectrophotometer, with measurements taken every 30 s for 3 min.

CAT was measured using the ultraviolet absorption method, with absorbance measured every 30 s at a wavelength of 240 nm at 25 °C, for a total of 3 min.

MDA was determined using the thiobarbituric acid (TBA) method, with a spectrophotometer measuring absorbance at 532 nm and 600 nm (using distilled water as a blank control).

(4) Determination of K^+^ and Na^+^ Ions:

K^+^ and Na^+^ were determined by dry ashing the sample, dissolving it in hydrochloric acid to a fixed volume, and measuring the absorbance at specific wavelengths (Na^+^ at 589.0 nm and K^+^ at 766.5 nm) using a flame atomic absorption spectrometer. The content was calculated based on the standard curve.

## 5. Conclusions

Attapulgite Clay-g-(AA-co-AAm) Hydrogel (ACH) has a loose, porous structure. As the addition of Attapulgite Clay increases (e.g., 5%), the surface roughness and porosity significantly improve. Under salt stress (NaCl), alkaline stress (NaHCO_3_), and mixed salt–alkaline stress (NaCl + NaHCO_3_), the addition of ACH significantly promotes spinach growth, particularly under severe salt stress (SS2), where 0.2% ACH increases leaf area by 91% and leaf weight by 95.69%. Additionally, ACH reduces proline content (up to 51.25% reduction under severe mixed stress) and MDA content (up to 68.98% reduction under severe alkaline stress), increases soluble sugar levels (up to 63.54% increase under mixed stress), and enhances antioxidant enzyme activity (SOD, POD, and CAT activity significantly increased under high stress), thereby strengthening the plant’s resistance. ACH also reduces Na^+^ accumulation in spinach roots and leaves (reducing by 61.12% in roots and 36.4% in leaves under severe salt stress), promotes normal K^+^ transport, and maintains “potassium–sodium balance.” Correlation analysis confirmed that ACH addition was positively correlated with leaf biomass and negatively correlated with proline, MDA, and leaf Na^+^ levels, indicating that it comprehensively alleviates the inhibitory effects of saline–alkali stress on spinach by synergistically regulating growth indicators, physiological metabolism, and ion homeostasis.

## Figures and Tables

**Table 1:** Experimental treatments in pots.

Treatment	ACH Addition	NaCl Addition	NaHCO_3_ Addition	Treatment	ACH Addition	NaCl Addition	NaHCO_3_ Addition
0%-CK	0%	0%		-	-	-	-
0.2%-CK	0.2%		0%	-	-	-	
0.4%-CK	0.4%			-	-	-	
0%-SS1	0%	0.2%		0%-SS2	0%		0%
0.2%-SS1	0.2%		0%	0.2%-SS2	0.2%	0.4%	
0.4%-SS1	0.4%			0.4%-SS2	0.4%		
0%-AS1	0%			0%-AS2	0%		0.4%
0.2%-AS1	0.2%	0%	0.2%	0.2%-AS2	0.2%	0%	
0.4%-AS1	0.4%			0.4%-AS2	0.4%		
0%-MS1	0%			0%-MS2	0%		
0.2%-MS1	0.2%	0.1%	0.1%	0.2%-MS2	0.2%	0.2%	0.2%
0.4%-MS1	0.4%			0.4%-MS2	0.4%		

## Data Availability

Data sharing does not apply to this article, as no datasets were generated or analyzed during the current study.

## Abbreviations

The following abbreviations are used in this manuscript:

ACH	Attapulgite Clay-g-(AA-co-AAm) Hydrogel
MDA	Malondialdehyde
SOD	Superoxide Dismutase
POD	Peroxidase
CAT	Catalase
